# Links between patient safety and fear of childbirth—A meta‐study of qualitative research

**DOI:** 10.1002/nop2.186

**Published:** 2018-07-16

**Authors:** Anne Lyberg, Bente Dahl, Megumi Haruna, Mizuki Takegata, Elisabeth Severinsson

**Affiliations:** ^1^ Department of Nursing and Health Sciences, Faculty of Health and Social Sciences, Centre for Women's, Family and Child Health University of South‐Eastern Norway Kongsberg Norway; ^2^ Department of Midwifery and Women's Health, Division of Health Sciences & Nursing Graduate School of Medicine The University of Tokyo Tokyo Japan; ^3^ Department of Paediatric Infectious Diseases, Institute of Tropical Medicine Nagasaki University Sakamoto Nagasaki Japan

**Keywords:** fear of childbirth, maternal trauma, motherhood, patient safety, professional support, qualitative meta‐study

## Abstract

**Aim:**

To conduct a meta‐study of qualitative empirical research to explore the links between patient safety and fear of childbirth in the maternity care context. The review questions were: How are patient safety and fear of childbirth described? and What are the links between patient safety and fear of childbirth in the maternity care context?

**Design:**

Meta‐study.

**Data sources:**

The CINAHL, Cochrane, PubMed, Webb of Science, Proquest and Medline (Ovid) electronic databases were searched for articles published between June 2000‐June 2016.

**Review methods:**

A meta‐study of qualitative research with a thematic analysis followed by a synthesis.

**Results:**

Four descriptive themes emerged: “Physical risks associated with giving birth vaginally”; “Control and safety issues”; “Preventing psychological maternal trauma and optimizing foetal well‐being”; and “Fear of the transition to motherhood due to lack of confidence”. The two overarching analytical themes: “Opting for safety” and “An insecure environment breeds fear of childbirth”, represent a deeper understanding and constitute the synthesis of the links between patient safety and fear of childbirth. This meta‐study indicates the need for increased commitment to safe care and professional support to reduce risks and prevent unnecessary harm in maternity care.

## 1 INTRODUCTION

The purpose of the World Health Organization (WHO) Patient Safety Programme is to facilitate the development of patient safety policy and practice in all member states and to act as a major force for improvement (WHO, 2008). Patient safety (PS) is intended to reduce risks and prevent unnecessary harm to patients as a result of healthcare (WHO, 2008). In recent years, the pursuit of increased safety for patients has led to a patient safety movement, especially in industrialized nations (Macchi, 2011). Kohn et al. (2000) recommended that healthcare organizations should create an environment where a safety culture is an explicit goal driven by leadership. Sammer, Lykens, Singh, Maines, and Lackan (2010) identified seven PS sub‐cultures: (a) leadership that acknowledges health care as high risk and seeks to align human resources; (b) teamwork characterized by collaboration on all levels in the organization; (c) patient care based on evidence‐based practices; (d) communication including an environment where an individual staff member has the right and responsibility to speak on behalf of a patient; (e) learning from mistakes and seeking opportunities for improvement; (f) recognizing errors as system failures rather than individual failures and at the same time not shrinking from holding individuals accountable for their actions and (g) care centred around the patient and her/his family. PS culture includes system, organizational and behavioural interventions, both individually and in combination. Teamwork training and improved communication with patients and between professionals are of the utmost importance for PS practice (Severinsson, Haruma, Rönnerhag, & Berggren, 2015). With regard to PS sub‐cultures in maternity care, it is likely that communication and woman‐centred care contribute to relieving fear of childbirth (FOC). According to Nilsson, Bondas, and Lundgren (2010), knowledge of women's needs and priorities is important, as positive birth experiences have a significant impact on the coping and well‐being of mother, child and family (Nilsson et al., 2010).

## 2 BACKGROUND

PS challenges concerning caesarean sections (CS) have been identified (WHO, 2015). While CS can be lifesaving for both the mother and unborn child, it is also used in situations where neither the mother nor the unborn child is at greater risk of complications than the rest of the peripartum population (Khunpradit et al., 2011). Reported CS rates vary, especially between developed and developing countries. In England, Scotland, Norway, Finland, Sweden and Denmark CS rates have risen from around 4% to 5% in 1970 to 20% to 22% in 2001 (Government Statistical Service (GSS) (2001); Macfarlane et al., 2000). In low and middle income countries, CS rates have also increased significantly during this period. Rates above 15% are reported in more than half of Latin American countries (Belizán, 1999). The overall CS rate in nine Asian countries was 27.3%. China had the highest CS rate, followed by Vietnam and Thailand (Lumbiganon et al., 2010). Betrán et al. (2016) conclude that the use of CS has increased to unprecedented levels. In 1985, the WHO issued a consensus statement suggesting that there were unlikely to be any additional health benefits associated with a CS rate above 10%–15%, while in 2015 the same organization described the increasing use of CS as a global health challenge and recommended vaginal birth as the first choice for healthy women (WHO, 2015). The main reasons for this recommendation are overuse of health resources and the risks involved in CS such as maternal infections, haemorrhage, the need for transfusion, injury to other organs, anaesthetic complications and psychological complications (International Cesarean Awareness Network (ICAN), 2002). Surveys conducted in Canada, the UK, Australia and Sweden have identified reasons for the increased number of CSs such as having undergone a previous CS, a negative birth experience and/or fear of giving birth (Edwards & Davies, 2001; Karlström et al., 2010; Pakenham, Chamberlain, & Smith, 2006; Waldenström, Hildingsson, & Ryding, 2006; Weaver, Statham, & Richards, 2007). The predominant reason for requesting a CS is FOC (Nieminen, Stephansson, & Ryding, 2009).

There has been a long‐standing focus on FOC in maternity care, but as it has been defined in various ways the literature on the subject is inconsistent (Zar, Wijma, & Wijma, 2002). The prevalence of FOC seems to depend on the definition of the condition, the measurement tools used and the cultural context. Previous population‐based studies in Scandinavia have found that FOC complicates 7.6%–17.8% of pregnancies (Laursen, Hedegaard, & Johansen, 2008; Nilsson, Lundgren, Karlström, & Hildinsson, 2012). The reasons for fear differ. Research on birth experiences and their association with fear has mostly focused on obstetric factors such as emergency CS, vacuum extraction and pain during labour (Størksen, Garthus‐Niegel, Vangen, & Eberhard‐Gran, 2008). Women's characteristics, such as anxiety, depression, low self‐esteem and lack of social support, have also been associated with FOC (Saisto & Halmesmäki, 2003). A connection has been found between FOC and a history of sexual assault, abuse and violence (Lukasse, Vangen, Øian, & Schei, 2010). Størksen et al. (2008) found that while obstetric complications do contribute to FOC, the association with previous subjective birth experiences is even greater and Walsh (2002) stated that the causes of FOC should be sought in maternity care rather than in the women's characteristics. Lyberg and Severinsson (2010) revealed that negative subjective birth experiences were often due to lack of a relationship with the midwife and other staff members, not being included in decision‐making, not having ownership of the birth and loss of dignity.

Subjective birth experiences are crucial from a PS perspective, while the objective characteristics of each birth and the woman's personality are less important. In the present study, the focus on PS and FOC also includes the first postnatal week as it is regarded as part of maternity birth services and during this period a sense of security is important for women's experiences of the transition to motherhood (Persson & Dykes, 2002). New mothers' physical and emotional experiences influence their well‐being (Waldenström & Rudman, 2008). Challenges concerning PS in the provision of quality maternity and postnatal care have been identified (Lyndon et al., 2015; Severinsson et al., 2015). Healthcare system users' experiences should be fundamental when assessing the quality of care (Berwick, 2002) and healthcare providers should be aware of what women need and want for a safe childbirth, as FOC is a problem for a significant number of women.

### 2.1 Aim

The aim was to conduct a meta‐study of qualitative empirical research to explore the links between PS and FOC in the maternity care context. The review questions were: How are PS and FOC described and What are the links between PS and FOC in the maternity care context?

## 3 METHODS

### 3.1 Design

A meta‐study approach inspired by Paterson, Thorne, Canam, and Jillings (2001) was employed. The analysis procedure involves three steps that should be undertaken prior to the synthesis. These are meta‐data analysis (the analysis of the findings) in a particular area; meta‐method (the analysis of methods) and meta‐theory (the analysis of the theory of the underlying structures on which the research is grounded) (Paterson et al., 2001, p. 10; Barnett‐Page & Thomas, 2009). The first step was to obtain an overview of the findings and analyse the substance of PS and FOC. The second step involved determining the methodological congruence of each article. We evaluated the sampling, data collection and analysis, as well as the data interpretation, rigor and auditability. In the third step, the links between PS and FOC were conceptualized. The three steps resulted in a synthesis that constitutes the meta‐study (Paterson et al., 2001, p. 13). Thus, a meta‐study is an interpretative qualitative approach to the phenomena of PS and FOC. The problems associated with understanding PS and FOC will be illuminated in the discussion.

### 3.2 Search strategy

**Figure 1:**
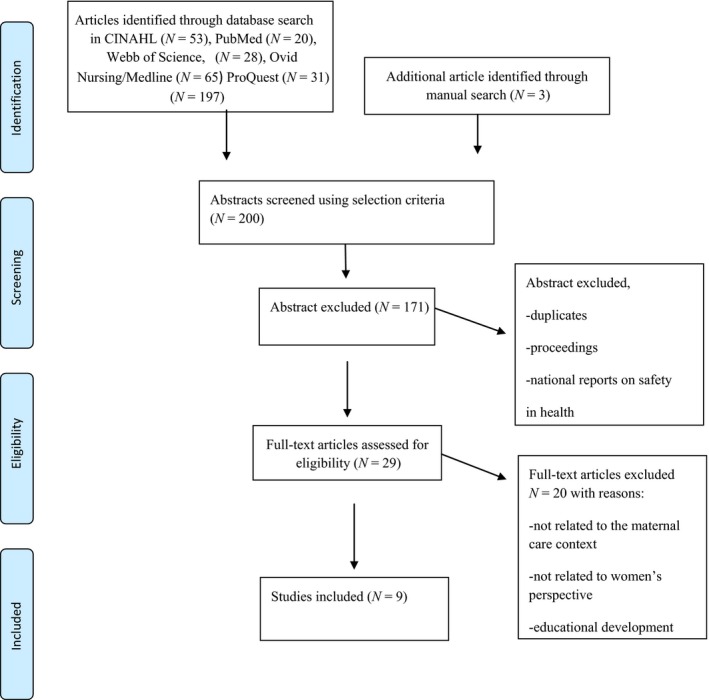
Data search using the PRISMA flow diagram (Moher et al., 2009)

A systematic search was conducted for articles published between June 2000‐June 2016 using the CINAHL, Cochrane, PubMed, Webb of Science, Proquest and Medline (Ovid) electronic databases. An example of the search terms employed in the CINAHL is: (MH "Patient Safety+") OR "patient safety" AND (MH "Obstetrics") OR “obstetrics” OR (MH "Labour+") OR “labour” OR “Labour” OR "Parturition" OR “Parturitions” OR (MH " Childbirth+)" OR "childbirth" OR "Childbirths" OR "Birth" OR "Births" OR (MH "Delivery, Obstetric+") AND (MH = “Fear+”) OR “fear” OR “fears” OR (MH = “Anxiety+” OR “anxiety” OR “Nervousness” OR “stress”). The inclusion criteria were: Articles about the concept, definition or description PS, PS practice and women suffering from FOC, how childbirth is organized, delivery of hospital care, assessment and care planning such as transitions to and from hospital settings and the first week of postnatal care. The reason for the time limit in the search was because the focus on patient safety issues was not very common in research before the year 2000 (Macchi et al., 2011). To achieve an understanding of the phenomena, it was decided to include studies based on qualitative research methods illustrating women's perspectives. The exclusion criteria were: Articles not related to the maternity care context, or women's perspective and studies involving quantitative research methods. The exclusion criteria are reported in Figure 1.

### 3.3 Eligible articles

The eligibility criteria for selecting articles were qualitative empirical studies focusing on the links between PS and FOC.

### 3.4 Search outcome

One hundred and ninety‐seven articles were identified before the elimination of duplicates. The selected articles were sorted by design, characteristics and location of the authors. During this process, three additional articles were identified through two manual searches, resulting in a total of nine empirical articles for analysis (Figure 1).

### 3.5 Critical appraisal of the included articles

The Joanna Briggs Institute (JBI), 2015) Critical Appraisal Tool for qualitative research (Lockwood, Munn, & Porritt, 2015) was used for quality assessment of the methodology. There are ten criteria for qualitative research: congruity between the stated philosophical perspective and the research methodology; congruity between the research methodology and the research questions or objectives; congruity between the research methodology and the methods used to collect data; congruity between the research methodology and the representation and analysis of data; congruity between the research methodology and the interpretation of results; the researchers' cultural beliefs and values that could potentially influence the study or theoretical orientation; influence of the researcher on the research and vice‐versa; representation of participants and their voices; ethical approval by an appropriate body; and relationship of the conclusions to the analysis or interpretation of the data.

Each article was assessed independently and subjected to rigorous appraisal by two of the researchers (A.L. and B.D.) to determine the quality to inform the synthesis and interpretation of the results. After a discussion on how to understand the questions in the Critical Appraisal Tool, the authors agreed on the appraisal of the included studies (Lockwood et al., 2015). There is an ongoing debate regarding the virtue of critical appraisal of qualitative studies during the review process. Lockwood et al. (2015) argue that appraisal is central to the credibility and transferability of qualitative evidence and for assisting and informing the reviewers in their decision about which studies to include in the review. No articles were excluded due to low quality (Appendix 1). All five researchers collaborated in the appraisal of the methodological characteristics and selection of the identified articles, while taking account of the review questions and links between the phenomena of FOC and PS.

### 3.6 Interpretative thematic synthesis

This study has an analytical explorative approach (Polit & Beck, 2012). Data extraction started by carefully reading and reflecting together on the content of the included studies to achieve a more comprehensive understanding and higher level of abstraction, the three stages presented by Thomas and Harden (2008, p.1) were used; line‐by‐line coding of the text to identify key concepts; the interpretation and development of descriptive themes; and the generation of analytical themes. The findings of each of the articles included in the review were interpreted to increase our understanding and move to another level of abstraction that goes beyond the individual findings. Hence the main findings were merged, transformed and interpreted against the background of the authors' pre‐understanding of maternity care and their professional experience as researchers, i.e. the first level of interpretation concerned the content of each study, while the second involved a comparison of the descriptive themes and components in their findings. Finally, on the third level, a pattern that illustrated the new insight was developed and discussed in relation to the two review questions. Two overarching analytical themes emerged that contribute to a deeper understanding and constitute the synthesis of the links between PS and FOC in maternity care. This stage of a qualitative synthesis is the most difficult to describe and also the most controversial, as it is dependent on the judgement and insight of the reviewers (Thomas & Harden, 2008). The whole analysis process involved joint reflection where the authors made use of their experience of working with qualitative data.

## 4 RESULTS

In total, nine articles were included and synthesized. Initially, four descriptive themes emerged in the analysis: (a) Physical risks associated with giving birth vaginally; (b) Control and safety issues; (c) Preventing psychological maternal trauma and optimizing foetal well‐being and (d) Fear of the transition to motherhood due to lack of confidence. In addition, two overarching analytical themes: “Opting for Safety” and “An insecure environment breeds fear of childbirth” emerged. Table 1 presents the included articles and their contribution to the results. In the following, the four themes and descriptions of the links between PS and FOC are presented.

**Table 1:** The list of included qualitative articles and their characteristics that contributed to the findings on the links between Patient Safety (PS) and Fear of Childbirth (FOC)

Authors/Year/Country	Aim	Method Participants/sample Data collection Analysis	Main findings about PS and FOC	Presence of key aspects in the articles contributing to the four interpreted themes describing FOC and PS			
				1	2	3	4^a^
Fenwick et al. (2010) Australia	To describe Australian women's request for caesarean section in the absence of medical indicators in their first pregnancy	Telephone interviews *n* = 14 women during their first pregnancy Theme‐based qualitative analysis	The women regarded vaginal birth as frightening, unpredictable and dangerous and therefore viewed a caesarean section as a more safe mode of delivery Women believed that the birth was unimportant and primarily about “getting” a baby	x	x	x	x
Forster et al. (2008) Australia	To understand women's views, expectations and experiences of early postnatal care	Eight focus group and four individual interviews *n* = 52 of whom seven were first‐time mothers Thematic network analysis	Women were concerned about the safety of their baby. The awareness of their responsibility for a new life contributed to anxiety and/or fear. There was a need for professional support in transition to motherhood as many lack confidence in their ability to care for the baby	‐	x	‐	x
Goberna‐Tricas et al. (2011) Spain	To investigate women's satisfaction with the quality of care and whether healthcare technology increases satisfaction or interferes with satisfaction in the process of care	Five focus groups with women attending the postnatal group programme The total number of participants and number of pregnancies are not mentioned Content analysis	Components of quality of care: safety, human dimension of the relationship between professionals and the patient and structural aspects that determine the context Safety is described as health technology and professional expertise with empathy and professional commitment	‐	x	x	‐
de Jonge et al. (2014) Netherlands	To study women who were referred during labour from primary to secondary care with regard to different aspects of continuity of care	Individual interviews *n* = 27 women with variation in parity Theme‐based qualitative analysis	Continuity of care was very important for feeling safe ‐Information gets lost during handover ‐Women wanted to be involved in decision‐making ‐fear of transportation during or after labour was reason for preferred hospital birth rather than home birth	x	x	‐	x
Larkin et al. (2012) Ireland	Exploration of women's experiences of childbirth	Five focus group interviews *n* = 25 women with variation in parity Theme‐based qualitative analysis	The birth process consists of phases which in different ways influenced the women's needs. In the article named as, getting started, getting there and consequences The context within which women give birth was important. Busy environments added to women's fears. They lost control and felt alone and unsupported	x	x	x	‐
Nilsson et al. (2010) Sweden	To describe the meaning of previous experiences of intense fear of childbirth	Individual interviews *n* = 9 women pregnant with their second child A phenomenological approach i.e., the essential structure of the phenomenon Qualitative analysis	The women reported a sense of not being present in the delivery room and an incomplete childbirth experience Having no control, meant that they felt confused and insecure with no place of their own A previous negative birth experience remained etched in the women's minds and gave rise to fear and lack of faith in their ability to give birth	x	x	x	x
Nilsson & Lundgren (2009) Sweden	To describe women's lived experience of fear of childbirth	Individual interviews *n* = 8 pregnant women seeking help for severe fear of childbirth. Two were pregnant for the first time (24–37 gestational weeks) Qualitative analysis	Feeling of danger, being trapped, being like an inferior mother‐to‐be and alone.	x	x	x	x
Persson et al. (2010) Sweden	To study factors that influence mothers' sense of security during their first postnatal week	Focus group combined with individual interviews Participants *n* = 14 all of whom were first‐time mothers Qualitative analysis	Postnatal sense of security was dependent on support, capacity and the health of the woman and her baby. Also having someone to turn to, knowing who to ask was important	‐	x	x	x
Petrovska et al. (2017) Australia	To examine the experiences of women who sought a vaginal breech birth to increase understanding of how to care for women seeking this birth option	Open‐ended questions focused on the methods used by women to source a clinician skilled in VBB^2^ *n* = not listed Thematic analysis	Stress, anger, fear and injustice were reported due to lack of support. System‐centred care versus woman‐centred care The challenge of shared decision‐making for vaginal breech birth	x	x	x	x

a Contributes to the following themes: 1) Risks associated with giving birth vaginally, 2) Control and safety issues 3) Preventing psychological maternal trauma and optimizing foetal well‐being 4) Fear of the transition to motherhood due to lack of confidence.

VBAC: vaginal birth after caesarean section; VBB: vaginal breech birth.

### 4.1 Physical risks associated with giving birth vaginally

This theme focuses on potential medical risks, complications and physical injury associated with vaginal birth. (Fenwick, Staff, Creedy, & Bayes, 2010; de Jonge, Stuijt, Eijke, & Westerman, 2014; Larkin, Begley, & Devane, 2012; Nilsson & Lundgren, 2009; Nilsson et al., 2010; Petrovska, Watts, Catling, Bisits, & Homer, 2017). Women regard CS as a safer, more appropriate way to give birth and reassigned the risks associated with CS (Fenwick et al., 2010). One important reason for women to request a CS was concern about physical injury as they underestimated the ability of their female body. They were also afraid of experiencing pain and undergoing specific interventions and procedures during labour (Fenwick et al., 2010; Nilsson & Lundgren, 2009). Furthermore, they considered it safer to protect the baby from the “stresses” of labour prior to a vaginal birth (Fenwick et al., 2010). Attitudes associated with childbirth are one of the challenges. The attitude that the birth was unimportant and primarily about “getting” a baby was reported (Fenwick et al., 2010). When women placed themselves in the hands of a surgical team for a CS, they relied on the professionals' high level of expertise to safeguard themselves and their child. A CS meant that the risk of a shortage of midwives to monitor the birth process and support the women was avoided (Larkin et al., 2012).

### 4.2 Control and safety issues

This theme focuses on control. The women considered that a CS was safe, calm and predictable (Fenwick et al., 2010). Larkin et al. (2012) reported that women's perceptions of control encompass a range of issues and contexts. The authors highlight information and the relationship with professionals as being of the utmost importance for the feeling of control. Being informed of the expected duration of labour and what will happen to the woman's body during labour helped the women. Satisfaction with care was reported when the hospital's technological facilities and healthcare professionals' technical expertise were available (Goberna‐Tricas, Banús‐Giménez, & Palacio‐Tauste, 2011). In contrast, the busyness of the hospital unit precluded woman‐centred care in early labour and in the period following the birth, thus some women stated that they would not have another baby due to their childbirth experiences as they considered that the maternity care professionals were not in control of the childbearing process (Larkin et al., 2012). Women were generally concerned about the safety of their baby (Forster et al., 2008). De Jonge et al. (2014) reported different PS management models and the need for continuity of care as the latter ensure a sense of safety and control.

### 4.3 Preventing psychological maternal trauma and optimizing foetal well‐being

This theme concerns maternal psychological trauma in addition to the well‐being of the mother and unborn child. Negative experiences with staff were reported by Nilsson and Lundgren (2009). The sense of not being present in the delivery room, not being allowed to actively participate in the birth and an incomplete childbirth experience remained etched in the women's minds, giving rise to fear (Nilsson et al., 2010).

Although midwives played an important role, it was suggested that they disempowered women and failed to promote positive experiences, leaving some women feeling alone and unsupported (Larkin et al., 2012). Two of the articles mention women's fear of maternal trauma due to diminished trust (Nilsson & Lundgren, 2009; Nilsson et al., 2010). A feeling of danger, being trapped and loneliness (Nilsson & Lundgren, 2009; Nilsson et al., 2010) as well as stress and fear (Petrovska et al., 2017) was reported.

### 4.4 Fear of the transition to motherhood due to lack of confidence

This theme focuses on the transition, a common theme in all nine articles. Most of the articles provide evidence of fear in relation to being unable to take responsibility for the new‐born baby in unpredictable situations (Fenwick et al., 2010), breastfeeding (Forster et al., 2008), lack of safety due to information getting lost during the handover and not being involved in decision‐making (de Jonge et al., 2014). It also includes the need to be encountered as an individual, receive relevant information, be prepared for the time after the birth, have someone to turn to, know who to ask and have planned follow‐up of the health of the mother and baby after discharge (Persson, Fridlund, Kvist, & Dykes, 2010).

### 4.5 Links between PS and FOC

Two overarching analytical themes represent a final synthesis of our understanding of the links between PS and FOC. The themes are intertwined and represent areas of inadequate PS practice on system, organizational and individual healthcare professional levels. The first, *Opting for safety*, indicates that women's knowledge of the risks of vaginal birth ensures that they take responsibility for themselves and the baby. PS practice is a guarantee of safe care and facilitates information about and an awareness of the potential risks of childbirth, as well as the responsibility inherent in becoming a mother. In addition, lack of control was reported (Fenwick et al., 2010; Foster et al., 2008; Goberna‐Tricas et al., 2011; de Jonge et al., 2014; Larkin et al., 2012; Nilsson & Lundgren, 2009; Nilsson et al., 2010; Person et al., 2010; Petrovska et al., 2017). Enhanced power, control and woman‐centred care are essential for a feeling of safety. The requests for a CS can be seen as a result of lack of communication with the midwife or lack of continuity with a trusted midwife. Decisions about the mode of birth are complex and our interpretation is that women try to minimize the risk by *opting for safety* as opposed to insecurity (de Jonge et al., 2014; Fenwick et al., 2010; Larkin et al., 2012; Nilsson & Lundgren, 2009; Nilsson et al., 2010; Petrovska et al., 2017). In addition, fear of the transition to motherhood was reported, implying that new mothers wanted to learn how to care for the baby (de Jonge et al., 2014; Fenwick et al., 2010; Forster et al., 2008; Nilsson & Lundgren, 2009; Nilsson et al., 2010; Persson et al., 2010; Petrovska et al., 2017).

The second analytical theme: “An insecure environment breeds fear of childbirth”, emphasizes the importance of understanding FOC. Like the previous analytical theme, it includes safety as well as lack of control in relation to the transition to motherhood due to lack of confidence (Fenwick et al., 2010; Forster et al., 2008; de Jonge et al., 2014; Larkin et al., 2012; Nilsson et al., 2010; Person et al., 2010). It can also be interpreted as a fear of complications, thus is linked to PS (Fenwick et al., 2010; Nilsson & Lundgren, 2009). Professional ability to detect when patients are at risk of harm is a prerequisite for managing unsafe situations, adverse events or near misses (Goberna‐Tricas et al., 2011; Larkin et al., 2012; Nilsson et al., 2010). One interpretation is that stressful situations may give rise to increased fear if the patient lacks trust in the healthcare professional (Forster et al., 2008; Goberna‐Tricas et al., 2011; Nilsson & Lundgren, 2009; Petrovska et al., 2017). Communication and teamwork problems are well known and a great challenge to PS.

## 5 DISCUSSION

The aim was to conduct a meta‐study of qualitative empirical research to explore the links between PS and FOC in the maternity care context. Four descriptive themes and two overarching analytical themes;

“Opting for safety” and “An insecure environment breeds fear of childbirth”, were identified, leading to an understanding of PS and FOC.

The two analytical themes can guide the continuous development of PS in maternity care. “Opting for safety” reveals the women's need to feel safe when giving birth and is, according to the studies included in this review, a challenge for maternity care. The relationship between FOC and previous birth experiences is described by Størksen et al. (2008) as well as Saisto and Halmesmäki (2003). Most women with FOC have negative birth experiences from a previous pregnancy, while many also have a “hereditary” fear due to stories told them by their mothers or friends (Sjögren & Thomassen, 1997). The synthesis of this study indicates that health care creates fear by not sufficiently addressing PS issues, especially interpersonal ones such as communication, shared decision‐making and teamwork. Interpersonal skills and professionality are aspects that can guide the development of maternity care. Lack of trust in the midwife and the need for enhanced power and control were common themes in the included studies. In many countries, maternity care has been centralized to a few busy hospitals, leading to a routinized care culture that fails to fulfil individual human needs (Berg, Ólafsdottir, & Lundgren, 2012). An example was found in a study from Sweden, where parents were: “waiting for permission to enter the labour ward world”, implying that parents made an effort to determine the appropriate time at which to arrive to avoid being refused entry for coming too early (Nyman, Downe, & Berg, 2011). It is likely that a positive first meeting and a welcoming atmosphere is of the utmost importance for the whole birth process. In our synthesis, FOC can be interpreted as a fear of surrendering. Although some women are empowered by their childbirth experience, others report feeling anxious, lonely and unsupported during and after the birth (Larkin et al., 2012). Midwives in modern institutional care are obliged to attend to more than one woman at a time, which could prevent them from being present in the delivery room to fulfil the women's need to be safeguarded (Larkin et al., 2012; Nilsson et al., 2010). This is in contrast to the midwifery model of woman‐centred childbirth care presented by Berg et al. (2012), where the importance of a reciprocal relationship between the midwife and the labouring woman and her partner is highlighted. A reciprocal relationship involves presence, affirmation, availability and participation. A midwife who is physically and mentally present is viewed as the essence of the encounter (Berg et al., 2012). In addition, Hunter, Lundgren, Ólafsdottir, and Kirkham (2008) claim that communication skills are the most important characteristic of a good midwife. One possible explanation for the increase in CS is that women choose this mode of childbirth when they lack trust in their midwife because they consider CS safer, more predictable and that it gives them a sense of control. Larkin et al. (2012) found that the continuous development of relationships with professionals either enhanced or detracted from the feeling of control. The length of postnatal hospital care has decreased in Scandinavian and many other countries in recent years. Researchers and policymakers are increasingly concerned about the low levels of satisfaction with hospital care following birth and have recommend that providers should give this area higher priority (McLachlan, Forester, Yelland, Rayner, & Lumley, 2008; Rudman & Waldenström, 2007). Brown, Small, Davis, Faber, and Krastev (2002) identified the most negative factors as the sensitivity of caregivers; the extent to which anxieties and concerns were taken seriously; how rushed caregivers seemed; the helpfulness of advice and support and whether help and support were offered at all. In our study, the transition to motherhood was found to be a complex process and women wanted to learn to care for their baby. Another concern in the postnatal period is raised by Munro, Janssen, Corbett, Bansback, and Kornelsen (2017), who reported that women start to reflect on future pregnancies and mode of delivery immediately after birth. In particular, women who regard the birth as unsafe and experienced a loss of control will construct birth as a frightening event. Such women need support to process the experience shortly after the birth, which is in line with Takegata et al. (2015) who argue for special attention for these women to help them cope with their childbirth experience in a more positive way. To develop PS in the context of maternity care, birth should be recognized to a greater extent as both a physical and an existential demand. Women are in need of a trusting relationship with healthcare professionals and time to recover in a supportive environment.

The second analytical theme in this synthesis was “An insecure environment breeds fear of childbirth”. Despite the development of technology and medical advances in maternity care, accidents, incidents and near‐misses still occur, thus safety concerns must be acknowledged to prevent harm (Martijn et al., 2013). A PS culture is characterized by open communication and a willingness to learn from adverse events (Severinsson et al., 2015). This contrasts with the study by Lyndon et al. (2015) on 3,282 physicians, midwives and registered nurses who care for women during labour and birth, where 90% of the respondents reported witnessing shortcuts, lack of competence, disrespect or performance problems in the preceding year. Although concerned about PS, they were not always willing to speak up and resolve these issues, the reason for which was the profound disagreements between professionals and providers about the resources and support necessary to deliver safe care. A sense of resignation regarding professionals' ability to change the situation was also found. These results indicate the need for healthcare organizations to create an environment where a safety culture is an explicit goal driven by leadership, as recommended by Kohn et al. (2000). The organization is responsible for providing PS and best practice guidelines. Although it is necessary to achieve an optimal care outcome for each woman, this does not necessarily correspond with healthcare system models. Overuse of ultra‐sound in addition to excessive monitoring of the unborn babies' heart rate and women's contractions are common. Maternity wards are designed to function effectively and equipped for medical interventions, which can lead to stress and feelings of insecurity for the woman and her partner. In her study, Nilsson (2014) found that midwives choose to follow medical routines rather than taking the women's needs into account, which gave the women a feeling of not being important and involved in the birth process. Another aspect of modern birth units at many hospitals is the installation of computer stations in each birth room. Foureur et al. (2010) found that the computers constitute an obstacle to effective, collaborative communication as the documentation routines are rigorous and distracting, resulting in less attention for the women. In turn, women can perceive the routines as uncaring, leading to feelings of being unseen and unprotected, which can result in a lack of confidence. On the other hand, the present findings reveal that many women also feel safe as a result of the high standard of technical equipment in delivery rooms. Nevertheless, Nilsson (2014) concludes that the delivery room is a place that creates FOC. As already mentioned, the duration and quality of postnatal care has decreased. While it is right for some women to leave the hospital after only a few hours, others need more time. The length of stay should be determined together with the woman, taking medical and psychosocial aspects into account. Women who choose early discharge should receive information about who to contact in the event of concerns about themselves or their baby. In many countries, there is a missing link in maternity services related to the first postnatal weeks. According to Carter et al. (2010), women, their baby and family should have access to adequate help that effectively addresses their mental, physical and social needs from the immediate postpartum period. When changes are made in health care, such as early discharge, new services must be developed to ensure PS.

The lack of confidence in maternity care delivery should be explored (WHO, 2015), which calls for leadership, innovation and integration of fundamental values, principles and goals to ensure safe, highly reliable individual practice (Carter et al., 2010). Woman‐centred care, continuity of care, teamwork and communication should be regarded as key components of an enhanced PS culture (Kohn et al., 2000) that may reduce feelings of insecurity and support each individual woman when giving birth.

### 5.1 Limitations

This meta‐study contributes to an increased understanding of the links between PS and FOC. However, some limitations should be discussed. To determine the transferability of a study, the range of empirical variation in the sample must be taken into consideration. In this study, the sample included women of different status and age from five countries. Although the number of studies included (*n* = 9) was fairly limited, it was considered appropriate as the findings from the analysis exceeded the results from the individual studies, thus enabling a synthesis. However, it is likely that PS and FOC may be understood differently in non‐Western cultures. PS may be commonly regarded as more “risk focused” by medical staff and mothers in countries with high CS rates, while the PS process including shared decision‐ making may differ in Asian societies where mothers tend to ask professionals to make a decision on their behalf. Furthermore, as healthcare professionals' working environment is culturally and socially sensitive, more evidence from other regions is required to make an optimal assessment of the cultural implications.

Credibility depends on the degree to which the study has explored the phenomenon it was intended to explore and if the methods used were appropriate. In this study, the data collection process and data results have been clearly described and are presented in Table 1. Although we conducted a broad literature search guided by an experienced librarian, we are aware that our choice of search terms and inclusion criteria may have affected the credibility.

The use of a critical assessment tool guided by a checklist deemed suitable for our purpose enabled a thorough overall appraisal of the articles and whether the methods employed were appropriate. All researchers read the papers and agreed on the themes, which involved collaborative work throughout the process. However, when conducting a meta‐study, data are decontextualized and removed from their original context, implying the risk that important findings in the primary research may be overlooked.

## 6 CONCLUSION

If the prerequisites for PS are lacking, it is likely that women will have little trust in maternity care. When women do not experience safety, they are afraid of giving birth and consider CS the preferred mode of delivery, which calls for attention. Feeling insecure in the first postnatal week also has a negative influence on the transition to motherhood. A PS culture is related to the systems and process of care. To achieve greater trust, we recommend educational interventions about the nature of PS to prevent incidents and FOC. This meta‐study indicates the need for increased commitment to safe care and professional support in order to reduce risks and prevent unnecessary harm in maternity care.

## CONFLICTS OF INTEREST

All authors declare that there are no conflicts of interest with regard to this study.

## AUTHORS' CONTRIBUTIONS

Design: A.L., E.S.; Manuscript preparation: A.L., B.D., E.S.; Conceptualization of the study: A.L., B.D., E.S.; Analysis and interpretation of the data: A.L, B.D., M.H., M.T., E.S. All authors have agreed on the final version and meet at least one of the following criteria recommended by the ICMJE (https://www.icmje.org/recommendations/)]:

- substantial contributions to conception and design, acquisition of data or analysis and interpretation of data
- drafting the article or critically revising it for important intellectual content

## APPENDIX 1

### Critical appraisal of the included studies by two authors working independently in accordance with the criteria (n = 10) of the Joanna Briggs Institute Critical Appraisal Tool for Qualitative Research (Y = yes; N = no; U = unclear; NA = not applicable)

**Table:**

Study	Author 1 (A.L.)										Author 2 (B.D.)									
	Q (Question)																			
	1	2	3	4	5	6	7	8	9	10	1	2	3	4	5	6	7	8	9	10
1 Fenwick et al. (2010)	Y	Y	Y	Y	Y	Y	N	Y	U	Y	Y	Y	Y	Y	Y	Y	N	Y	Y	Y
2 Forster et al. (2008)	Y	Y	Y	Y	Y	Y	N	Y	Y	Y	Y	Y	Y	Y	Y	Y	N	Y	Y	Y
3 Goberna‐Tricas et al. (2011)	N	Y	Y	Y	Y	Y	N	Y	N	Y	N	Y	Y	Y	Y	Y	N	Y	N	Y
4 de Jonge et al. (2014)	Y	Y	Y	Y	Y	Y	N	Y	Y	Y	Y	Y	Y	Y	Y	Y	N	Y	Y	Y
5 Larkin et al. (2012)	Y	Y	Y	Y	Y	Y	N	Y	U	Y	Y	Y	Y	Y	Y	Y	N	Y	U	Y
6 Nilsson et al. (2010)	Y	Y	Y	Y	Y	Y	N	Y	Y	Y	Y	Y	Y	Y	Y	Y	N	Y	Y	Y
7 Nilsson & Lundgren (2009)	Y	Y	Y	Y	Y	Y	N	Y	Y	Y	Y	Y	Y	Y	Y	Y	N	Y	Y	Y
8 Persson et al. (2010)	Y	Y	Y	Y	Y	Y	N	Y	Y	Y	Y	Y	Y	Y	Y	Y	N	Y	Y	Y
9 Petrovska et al. (2017)	Y	Y	Y	Y	Y	Y	N	Y	U	Y	Y	Y	Y	Y	Y	Y	N	Y	U	Y
